# Characterization, diversity, and biogeochemical potential of soil viruses inhabiting in Yuncheng Salt Lake

**DOI:** 10.3389/fmicb.2025.1597514

**Published:** 2025-05-07

**Authors:** Jin Liu, Xiaoxiao Dong, Xiaokai Wang, Yunmeng Chu

**Affiliations:** ^1^Department of Life Sciences, Yuncheng University, Yuncheng, Shanxi, China; ^2^National Key Laboratory of Crop Genetic Improvement, Huazhong Agricultural University, Wuhan, Hubei, China; ^3^01Life Institute, Shenzhen, Guangdong, China; ^4^Shenzhen Institutes of Advanced Technology, Chinese Academy of Sciences, Shenzhen, Guangdong, China

**Keywords:** salt lake, soil virus, characterization, diversity, viral auxiliary metabolic gene

## Abstract

Viruses play a crucial role in microbial communities and can significantly influence ecosystem processes and biogeochemical cycles by regulating the structure of these communities and the metabolic functions of their hosts. Salt lakes are recognized for harboring a diverse array of halotolerant microorganisms; however, there is limited knowledge regarding the viruses and their co-occurring hosts in these halotolerant environments. Herein, 3,362 viral operational taxonomic units (vOTUs) were recovered from Yuncheng Salt Lake soils by combining with a binning method, with less than 2% showing homology to known viruses, highlighting a significant unexplored viral diversity. Virus communities were typically specific to their habitats, exhibiting low overlap across various geographical regions and other ecological environments. It was predicted that 27.4% of viruses were associated with microbial host populations, and the majority of the predicted vOTUs are linked to dominant bacteria and archaea. Metabolic predictions revealed that 568 putative auxiliary metabolic genes (AMGs) were involved in various processes related to biosynthesis and transformation reactions associated with resource utilization within host cells. The virus-encoded AMGs indicated that these viruses influenced the metabolism of carbon, nitrogen, sulfur, and phosphorus in microorganisms, as well as their adaptation to salinity. This study highlighted the unique characteristics and potential ecological roles of soil viruses in Yuncheng Salt Lake, suggesting that these viruses may significantly influence microbial communities and the biogeochemistry of salt lake soils. These findings provide valuable insights into the diversity, function, and ecology of viruses in soils surrounding salt lakes, establishing a foundation for understanding their roles in these unique ecosystems.

## 1 Introduction

A salt lake is a saline water body with a salinity greater than 50 g/L. It forms through a natural process that involves the continuous influx of saline water and the influence of geological conditions, leading to the accumulation of complex substances, such as salts (Lindsay et al., 2019). Collectively, salt lakes have an estimated volume of approximately 1.04 × 10^5 km^3^, which is comparable to the volume of freshwater lakes, estimated at around 1.25 × 10^5 km^3^ (Huang et al., 2023). Salt lakes are distributed globally, accounting for approximately 44% of the total water volume of all lakes and 23% of the overall surface area on the planet (Huang et al., 2023). Microorganisms that can endure high salinity thrive in these environments, where elevated salt concentrations inhibit the survival of many other organisms (Zeng et al., 2022). The complexity of these microorganisms, along with their unique metabolic processes and ability to withstand extreme conditions (such as low temperatures and high salinity), makes them valuable biological resources in extreme environments, offering significant potential for practical applications. In summary, typical salt lakes exihibit a range of gradients, including varying salinity, pH, and high altitudes (Zheng and Liu, 2009; Yang et al., 2016). These characteristics provide a unique natural laboratory for studying microbial diversity and their adaptations to environmental changes.

Yuncheng Salt Lake, located on the southern edge of Yuncheng City in Shanxi Province, China, is often called the “Dead Sea of China” (Zhang et al., 2020). It is recognized as the third-largest natural inland sodium sulfate lake in the world, situated between 110°07′ to 110°50′E and 34°04′ to 34°54′N. The lake is rich in mirabilite, salt, and various minerals, making it an important reservoir of mineral and biological resources (Gao et al., 2021). However, rapid social and economic development has led to the over-exploitation of these resources, causing substantial ecological degradation. A prolonged lack of ecological water has further contributed to the lake’s shrinkage, threatening the biological populations necessary for ecological balance. In response, researchers have investigated Yuncheng Salt Lake (Zhang et al., 2019; Zeng et al., 2022), focusing on the human environment, salt-tolerant flora (Gao et al., 2021), halotolerant microorganisms (Kanik et al., 2020; Ming et al., 2020), and salt production (Zhou et al., 2019; Ming et al., 2020; Gao et al., 2021). However, significant gaps remain in the research regarding the genomic diversity of viruses in Yuncheng Salt Lake and their potential ecological roles in biogeochemical cycles. Considering the distinctive salinity conditions of Yuncheng Salt Lake, it promotes a unique diversity of microorganisms and viruses. Further exploration of the diversity and structure of viral communities, as well as their potential ecological functions in biogeochemical cycles, is essential.

Viruses, as the most abundant biological entities on Earth, are non-cellular microorganisms that rely on host cells for replication (Ji et al., 2021). They play critical roles in various ecosystems—aquatic, terrestrial, and human-associated—significantly influencing the structure and functioning of microbial communities (Paez-Espino et al., 2016). By infecting and lysing host cells, viruses alter the dynamics of microbial communities and release a large amounts of metabolic substances for other microorganisms to utilize (Suttle, 2005; Mayers et al., 2023). In addition, viruses as important carriers of horizontal gene transfer, can regulate metabolic processes in ecosystems by transferring genes that encode metabolic functions (Jansson and Wu, 2023). Researches have shown that viruses can acquire auxiliary metabolic genes (AMGs) from their hosts and transfer these genes in and out of various host cells along with the viral particles (Emerson et al., 2018; Jin et al., 2019; Mara et al., 2020). When the virus is in a lysogenic state, the expression of these host-derived genes can “hijack” or enhance host metabolic processes (Warwick-Dugdale et al., 2019), thereby contributing to ecosystem functioning by encoding enzymes essential for biogeochemical transformations. These genes can also expand the environmental niches accessible to their hosts (Sun et al., 2023). Notable examples of AMGs include genes related to photosynthesis (Millard et al., 2004; Thompson et al., 2011), as well as those involved in nucleotide metabolism and the cycling of nitrogen, phosphorus, sulfur, and various carbon metabolic processes, including methane oxidation (Sun et al., 2023).

This study aimed to investigate the characterization, geographical distribution pattems, and biogeochemical potential of viruses in salt lake soils. We analyzed 48 soil metagenomes from various sites around Yuncheng Salt Lake. The recovery of the Salt Lake viral genomes revealed unique geographical distribution characteristics, detailing the diversity of viral entities, their associations with hosts, and the presence of virus-encoded AMGs. Our findings contribute hundreds of novel viral genomes from salt lake soils to the expanding catalog of known viruses, thereby enhancing our understanding of viral diversity and functionality in extreme soil ecosystems.

## 2 Materials and methods

### 2.1 Metagenomic collection and assembly

The soil samples were collected from Yuncheng Salt Lake, which is located in Shanxi Province. Soils were collected from the southeast (SE), northeast (NE), southwest (SW), and northwest (NW) directions at sites located 15 m, 30 m, 45 m, and 60 m from the salt lake in each direction. The total DNA was extracted from soil samples using the Magnetic Soil and Stool DNA Kit (DP712, TIANGEN, China), following the manufacturer’s instructions. For metagenomic sequencing, a paired-end library was constructed from the total DNA using the NEBNext Ultra DNA Library Prep Kit (NEB, USA) and sequenced on an Illumina NovaSeq 6,000 platform. Detailed information regarding the study sites and sampling methods has been documented in previous research (Zeng et al., 2022). The raw reads were trimmed and quality-filtered using Fastp v0.20.1 (Chen, 2023) to generate high-quality clean reads. Then, the clean reads were assembled into contigs using SPAdes v3.15.5 (−meta) (Bankevich et al., 2012), and short contigs smaller than 1 kb were filtered out.

### 2.2 Prokaryotic metagenome-assembled genome generation

Contigs from each assembly were binned with parameters --maxbin2, −-metabat1, and --metabat2 in metaWRAP (Uritskiy et al., 2018), then consolidated into a final bin set with the Bin_refinement module (−c 50, −x 10). For the generated bin set, utilized dRep v2.3.2 (−comp 50, −con 10, −sa 0.95) (Olm et al., 2017) to cluster and dereplicate at a 95% average nucleotide identity (ANI) threshold, resulting in 592 species-level metagenome-assembled genomes (MAGs). Taxonomic classification was conducted with GTDB-Tk v1.6.0 (Chaumeil et al., 2020; Parks et al., 2020), and genome completeness and contamination were assessed using CheckM v1.1.3 (Parks et al., 2015) with default parameters. The dataset, which consists of 592 prokaryotic MAGs, was used to predict viral hosts.

### 2.3 Identification of viral sequences

The procedures and criteria for viral sequence identification were outlined as follows: (1) VIBRANT v1.2.1 with the “-virome” parameter to enhance candidate viral sequence identification (Kieft et al., 2020); (2) VirSorter v2.2.3 (Guo et al., 2021), which classified sequences as high-confidence viruses based on a score of ≥ 0.9 or a score between 0.75 and 0.9 with at least one viral gene; and (3) CheckV v0.8.1 (Nayfach et al., 2021), which identified viral sequences based on the presence of at least one viral gene. Merged the putative viral contigs and eliminated any duplicates. Utilized vRhyme v1.1.0 (Kieft et al., 2022) with default parameters to bin the viral contigs into viral metagenome-assembled genomes (vMAGs), thereby enhancing the completeness of the viral genomes. Following established protocols, putative viruses (≥ 10 kb) were selected from the vMAGs and unbinned viral contigs. For putative viruses shorter than 10 kb, complete or circular viral sequences were retained based on assessments from CheckV, VIBRANT, and vRhyme. The viral sequences were compiled and subsequently clustered using CD-HIT v4.8.1 (parameters: ≥ 95% identity and ≥ 85% coverage), resulting in 3,362 viral populations, or viral operational taxonomic units (vOTUs), for downstream analyses in accordance with the previously suggested pipeline (Roux et al., 2019b). Finally, the completeness and lifestyle of the 3,362 vOTU sequences were estimated using the CheckV pipeline (Nayfach et al., 2021). Using BLASTn (Altschul et al., 1997) (E value < 1e-5, identity ≥ 90%, alignment length ≥ 1 kb) to compare our vOTUs with the IMG/VR v4 database (Camargo et al., 2023) to evaluate the novelty of these viruses.

### 2.4 Taxonomic assignments and abundance profiles of viruses

Two methods were used to assign family-level taxonomic annotations to viral sequences. The first method involved gene-sharing network analysis using vConTACT2 v0.9.19 (Jang et al., 2019) with Viral RefSeq database (v211). This approach enabled the assignment of the viral sequences to known taxonomic genera and families. The second method utilized a previously established majority-rules approach (Gregory et al., 2019). Specifically, we implemented the Demovir pipeline^1^ with default parameters (Shkoporov et al., 2019; Liao et al., 2022) to identify sequence homologies between virus-encoded proteins to a redundant viral subset of the TrEMBL database. When more than 50% of the proteins in a viral sequence belonged to the same family, the virus was classified as a member of that family (Liao et al., 2022).

### 2.5 Relative abundance profiles of viruses

Clean reads of each soil sample were mapped to the 3,362 vOTUs using Bowtie2 (Langmead and Salzberg, 2012) to calculate viral relative abundance profiles. The resulting SAM files were processed with SAMtools (Li et al., 2009), and then the sorted BAM files were analyzed using CoverM v0.3.1^2^ to generate Reads Per Kilobase per Million Mapped Reads (RPKM) profiles for all samples (Yu et al., 2024).

### 2.6 Phylogenetic analysis of terL gene

A phylogenetic tree illustrating the viral diversity in Salt Lake soils was constructed using the gene that encodes the terminase large subunit (*terL*), a hallmark of *Caudovirales* (tailed dsDNA phages) (Cheng et al., 2022; Chu et al., 2022). The Pfam 33.1 database (Mistry et al., 2021) were used to extract the TerL protein domain from vOTUs in this study by HMMER3 (Söding, 2005). The TerL proteins of viral RefSeq were obtained from the NCBI Virus database^3^ in August 2024. All TerL proteins were combined and aligned these sequences using MUSCLE v3.8.31 (Edgar, 2004), and gaps were trimmed using trimAl v.1.4 (−gappyout) (Capella-Gutiérrez et al., 2009). Used IQ-TREE v1.6.12 (Nguyen et al., 2015) with the model (−m LG + R4) (Chu et al., 2022) for phylogenetic reconstruction, and manually edited the phylogenetic tree using iTOL (Letunic and Bork, 2019).

### 2.7 Comparison to viruses from other data sets

We conducted a comparative analysis of viral sequences from Yuncheng Salt Lake soils against similar environments and the RefSeq database through a protein-sharing network analysis. This included saline water viruses (*n* = 19,709) and marine sediment viruses (*n* = 3,317) from the IMG/VR v4 database (Camargo et al., 2023). Used Prodigal v2.6.3 (Hyatt et al., 2010) with the “-p meta” option to predict open reading frames (ORFs) and performed all-to-all BLASTp comparisons of proteins using DIAMOND v2.0.15 (Buchfink et al., 2015). vConTACT2 v0.11.3 (Jang et al., 2019), in conjunction with the Viral RefSeq database (v211), was used to cluster viral genomes into viral clusters (VCs) based on protein-sharing networks.

### 2.8 Prediction of virus and host linkages

The linkages between vOTUs and putative host MAGs were established using four *in silico* methods, as previously reported (Emerson et al., 2018; Li et al., 2021; Chu et al., 2022). These methods were based on the following ranked criteria (Emerson et al., 2018; Luo et al., 2022): (i) CRISPR spacer matches. CRISPR spacers of putative host MAGs were extracted using CRISPRCasFinder v4.2 (Mitrofanov et al., 2021), and BLASTn was used to compared these CRISPR spacers to vOTU sequences (parameters: e-value ≤ 1e-5, identity ≥ 95% and mismatches ≤ 2) (Emerson et al., 2018). (ii) Transfer RNA (tRNA) homology. tRNA sequences in vOTUs were retrieved using tRNAscan-SE v2.0.9 (Chan et al., 2021) and run against to MAGs via BLASTn (parameters: identity ≥ 90%, coverage ≥ 90%) (Fan et al., 2023). (iii) Nucleotide sequence homology. vOTUs were aligned to MAGs using BLASTn, and matches with ≥ 70% identity and aligned length ≥ 2 kb were retained (Roux et al., 2016). (iv) Oligonucleotide frequency (ONF) similarity. The ONF-based distances of vOTUs and MAGs were calculated using VirHostMatcher v1.0.0 (Ahlgren et al., 2017) with default parameters, and matches with d2* values of ≤ 0.2 were retained (Li et al., 2021; Chu et al., 2022). If multiple hosts of a specific vOTU were predicted by multiple methods, the virus-host linkages were retained (Gios et al., 2024).

### 2.9 Functional profiles and identification of AMGs

Gene annotations were performed for all proteins of 3,362 vOTUs using eggNOG-mapper v2.085 (Cantalapiedra et al., 2021) with the eggNOG database v5.0 (Huerta-Cepas et al., 2019), with the parameters: e-value ≤ 1e-5, bit score ≥ 50. The functions of these viral genes were classified according to COG functional categories. Viral AMGs were predicted for vOTUs using two methods: (i) the DRAM-v1.4.6 pipeline (Shaffer et al., 2020). VirSorter2 (−-prep-for-dramv) were used for vOTU sequences to generate input files for DRAM-v. In the results of DRAM-v, AMG annotations with auxiliary scores < 4 were retained. (ii) The VIBRANT v1.2.1 pipeline was used, where vOTUs were inputted to identify AMGs and provide annotations, with default parameters. Combined the AMGs predicted based on the above pipeline, manual curations were performed to remove false positive AMGs (such as nucleotide metabolism, organic nitrogen, glycosyltransferases, and ribosomal proteins), as previously reported (Li et al., 2021; ter Horst et al., 2021; Chu et al., 2022). Putative AMGs located between or alongside two viral hallmark genes or virus-like genes were considered as high-confidence viral AMGs for further analysis (Pratama et al., 2021).

### 2.10 Statistical analyses

All statistical analyses were conducted using R v4.2.1. Diversity indices, including Shannon, Simpson, Chao1, and PCoA (utilizing the Bray–Curtis dissimilarity metric) were calculated with the vegan (v.2.5–7) package. Data visualizations were created in ggplot2, with Sankey plots generated via ChiPlot.^4^

## 3 Results and discussion

### 3.1 Soil viruses around salt lake

**Figure 1:**
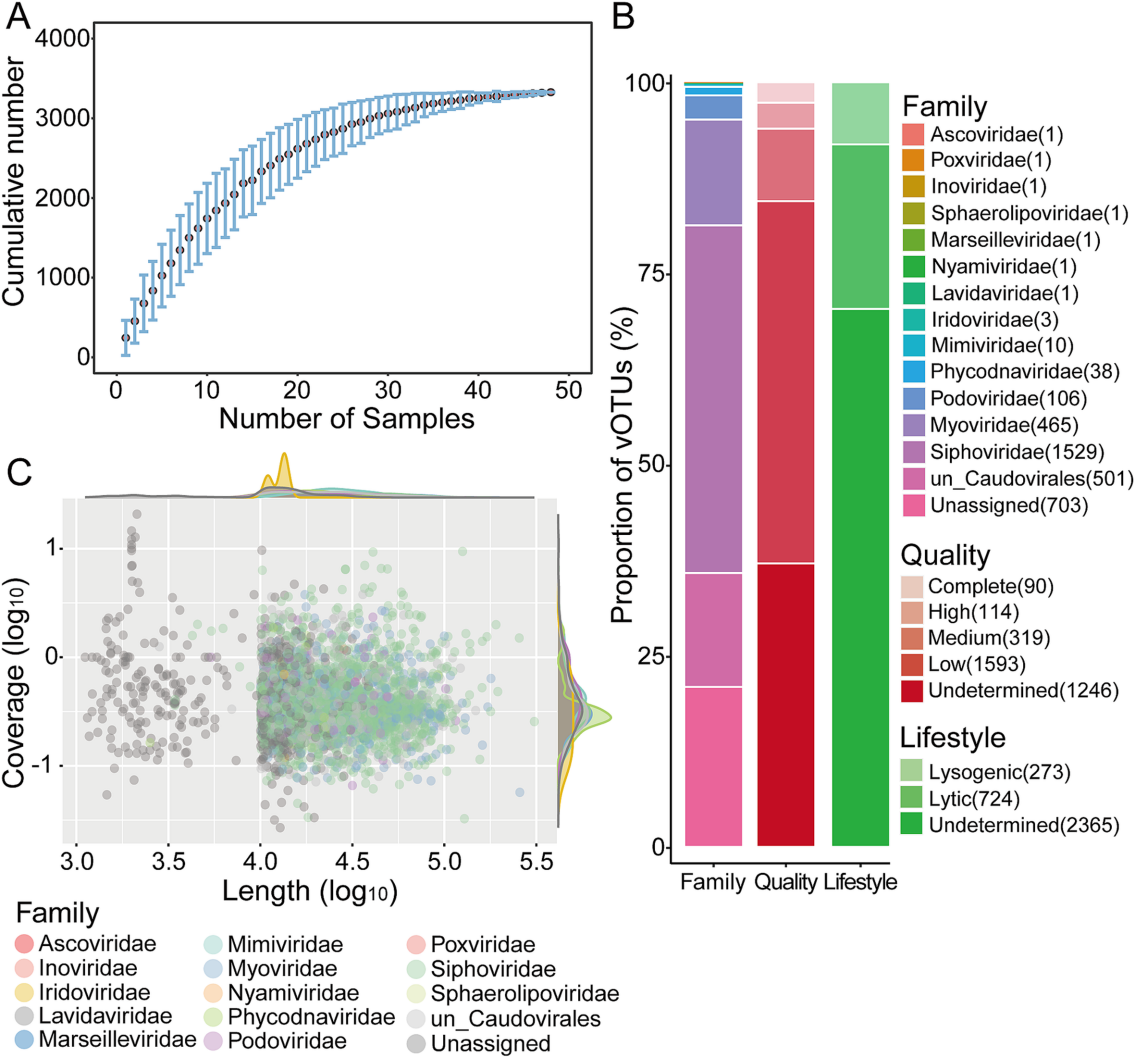
Overview of soil viruses around Yuncheng Salt Lake. **(A)** Accumulation curve of vOTUs in this study. Dots represent the average number for all combinations of a given number of samples, and error bars represent the range. **(B)** Bar charts showing the quality, lifestyle and taxonomy of the Salt Lake soil viruses. **(C)** Distribution of genome size and coverage of viruses. Each circle represents one viral sequences.

The metagenomes of Yuncheng Salt Lake soils yielded 76,847 putative viral contigs, which were analyzed using three software tools to identify viral sequences. To reconstruct longer and more complete genomes, these contigs were binned with vRhyme and resulting in 4,820 vMAGs. Then, 57,237 putative viral sequences including unbinned contigs and these vMAGs were manually filtered and clustered to approximate species-level taxonomy (Gregory et al., 2019; Ma et al., 2024). Finally, this process produced 3,362 non-redundant vOTUs, comprising 2,951 vMAGs and 411 viral contigs, each with a length ≥ 10 kb or represented by circular/complete sequences. The saturation cumulative curve of Salt Lake vOTUs indicated that the viral communities present in these soils were relatively well-sampled (Figure 1A). Among the 3,362 vOTUs, 95% exceeded 10 kb, while 180 vOTUs were shorter. The lengths of the vMAGs ranged from 4.27 kb to 308 kb, with most viral sequences exceeding 10 kb, although seven circular/complete genomes fell below this threshold. Three vMAGs longer than 200 kb were classified as “huge phage” based on the presence of phage-specific genes (Al-Shayeb et al., 2020). The quality and completeness of the 3,362 vOTUs were assessed using CheckV. It classified approximately 63% into four quality tiers: complete genomes (90 vOTUs; 100% completeness), high-quality genomes (114 vOTUs; >90% completeness), medium-quality genomes (319 vOTUs; 50–90% completeness), and low-quality genomes (1,593 vOTUs; <50% completeness). The completeness of 1,246 vOTUs (37%) could not be determined (Figure 1B; Supplementary Table S1). The proportion of high-quality viral genomes in Salt Lake soils was only 6%, whereas it reached 9% in the Challenger Deep oceanic trench (Zhou et al., 2022), even without binning to improve viral genome completeness. Possible reasons for this difference include, on the one hand, the low abundance of microbial communities in salt lake soils, on the other hand, insufficient metagenomic sequencing. The completeness of the viral genomes may be improved by increasing sequencing depth or by employing HiFi metagenomic sequencing (Tao et al., 2023).

Of the 3,362 vOTUs, 79% were successfully classified (Figure 1B; Supplementary Table S1), and genome length and coverage of these identified viral sequences were shown in Figure 1C. Most soil viral sequences belonged to the order *Caudovirales*, accounting for 79.08% (*n* = 2,601) of the total. This included the families *Siphoviridae* (*n* = 1,529, 42.5%), *Myoviridae* (*n* = 465, 13.8%), and *Podoviridae* (*n* = 106, 3.2%). The remaining sequences (*n* = 501, 14.9%) were classified to *Caudovirales* but did lacked specific family classification. *Caudoviricetes* are characterized as double-stranded DNA (dsDNA) tailed phages, which typify most viruses that infect bacteria and archaea (Ackermann and Prangishvili, 2012). These viral families have been well-documented in deep-sea sediments (Li et al., 2021; Zhou et al., 2022; Yu et al., 2024), global soil environments (Ma et al., 2024), and saline lake ecosystems (Gu et al., 2021; Tao et al., 2023; ZeinEldin et al., 2023). Additionally, nucleocytoplasmic large DNA viruses (NCLDVs) that infect eukaryotic organisms were identified in Salt Lake soils, including *Phycodnaviridae* (*n* = 38, 1.13%), *Mimiviridae* (*n* = 10, 0.30%), and *Iridoviridae* (*n* = 3, 0.09%), along with other families such as *Marseilleviridae*, *Ascoviridae*, and *Poxviridae*. *Lavidaviridae* that are “virophages,” classified as parasites of giant eukaryotic viruses (Roux et al., 2023), were also detected. The relative abundances of NCLDVs and virophages were notably low, each comprising less than 1%, consistent with previous findings in the virome of salt lakes on the Qinghai-Tibet Plateau (Gu et al., 2021). Single-stranded DNA (ssDNA) viruses (*Inoviridae*) and RNA viruses (*Nyamiviridae*) were also found in Yuncheng Salt Lake soils, these viruses have been previously identified in oceanic environments, including deep-sea sediments (Yu et al., 2024). However, their numbers and categories remain limited, and their diversity and function warrant further investigation. It should be noted that some DNA viruses may contain RNA virus marker genes, such as the RNA-Dependent RNA Polymerase (RdRp) gene, which can result in their misclassification as RNA viruses. Consequently, few RNA viruses may be detected in DNA-based metagenomic data. Yu et al. reported the identification of several RNA virus families within deep-sea metagenomic data (Yu et al., 2024).

To assess the lifestyle of viral sequences in Salt Lake soils, potential temperate viruses typically present as prophages or viral sequences within integrase-encoding genes, were predicted from all vOTUs using VIBRANT with the KEGG, Pfam, and VOG databases (Kieft et al., 2020). Our findings revealed that 2,365 vOTUs, representing 70% of the viral communities in Salt Lake soil, could not be classified as either lytic or lysogenic (see Figure 1B; Supplementary Table S1). A significant proportion of these unclassified viral contigs may be associated with viruses exhibiting a lytic lifestyle in Yuncheng Salt Lake (Wang and Li, 2018; Wang et al., 2022). This observation aligns with previous studies of viral communities in saline lakes on the Qinghai-Tibet Plateau, which reported elevated rates of viral lysis (Gu et al., 2021). Lysogeny was predicted in only 8% (273 out of 3,362) of the Salt Lake soil viruses, contrasting with sediments of Baltic Sea (approximately 19%), where lysogeny appears more prevalent (Cai et al., 2019). Nevertheless, this finding is consistent with predictions from deep-sea sediments (~7%) (Zhou et al., 2022; Yu et al., 2024) and saline soil viruses (7%) (Gu et al., 2021) as determined by VIBRANT. It is important to interpret our conclusions cautiously, given that 70% of the viral contigs remain unclassified as either lytic or lysogenic.

### 3.2 Novelty of salt lake soil viruses

The majority of viruses identified in Yuncheng Salt Lake soils had not been uncharacterized previously. Specifically, consistent with prior reports (Gu et al., 2021), analysis showed that 98.69% (3,318 out of 3,362) of the vOTUs were novel, with no homologs in the IMG/VR 4 dataset, which includes various habitats (Camargo et al., 2023). Of the 44 viral sequences with homologs, 4 were classified as unclassified *Caudovirales*, and 4 had no taxonomic classification. These results highlight the distinctive characteristics of the virome observed in this study, as well as the limitations inherent in virome research conducted in salt lakes.

**Figure 2:**
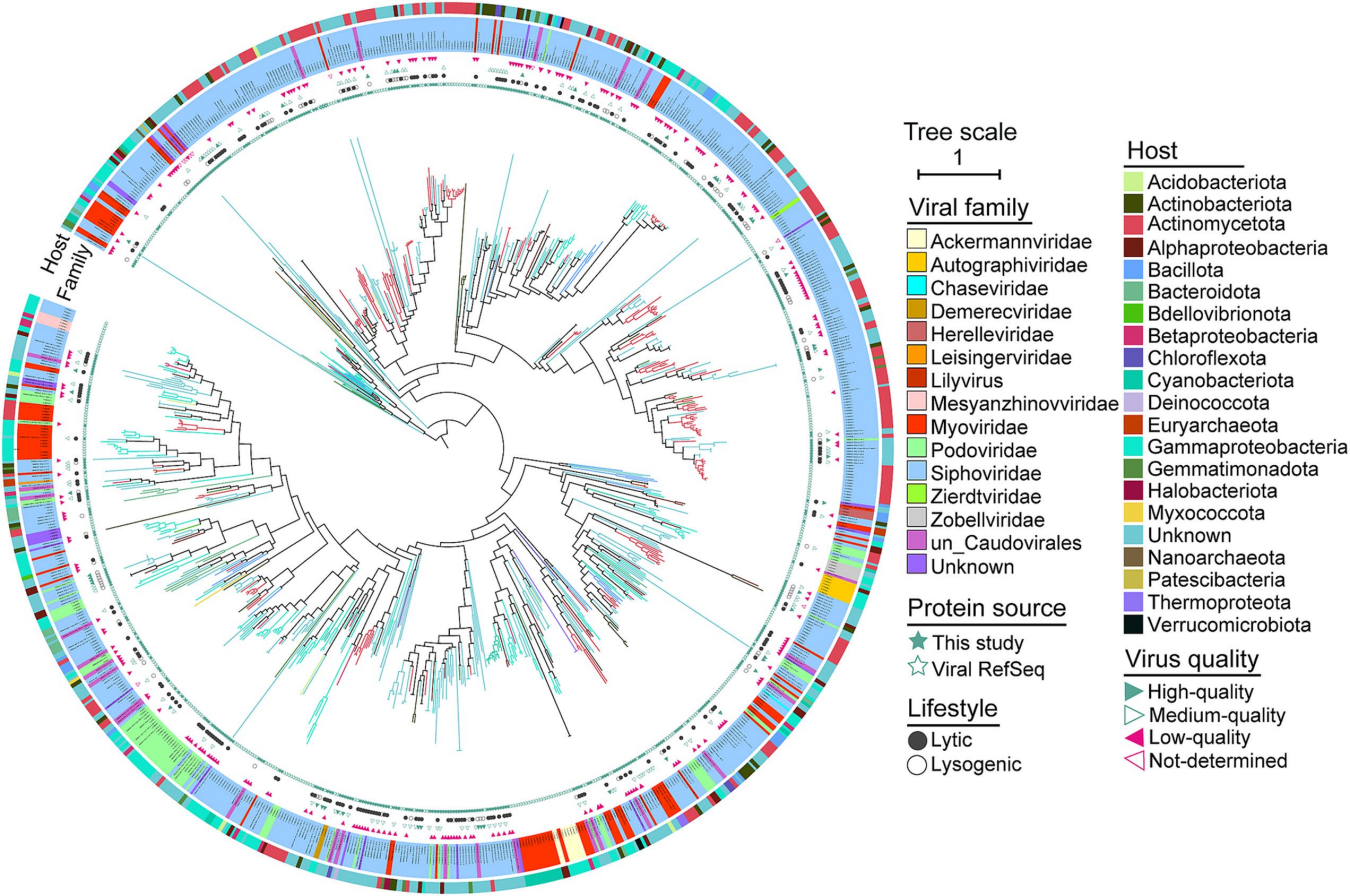
Phylogenetic tree of the *Caudovirales* viruses in soils. A maximum-likelihood tree was constructed using TerL proteins of *Caudovirales*. Clades and the names are colored according to viral family. The reference genomes and Salt Lake soil viruses (this study) are presented in soild pentacle and hollow pentacle, respectively. Lifestyle and quality of viruses are in presented in circles and triangles, respectively. The outermost ring show the viral host phyla (classes for *Proteobacteria*).

The *terL* gene, as a hallmark that is frequently used for phylogenetic reconstruction of *Caudovirales* phages and appears to be the best phylogenetic marker due to its widespread presence among phages and its high level of sequence conservation (Low et al., 2019; Barylski et al., 2020). Therefore, we constructed phylogenetic trees using TerL protein sequences to assess the evolutionary distance among bacteriophages in Salt Lake soils. We identified 413 complete ORFs encoding TerL proteins in 413 viruses (413/3362 vOTUs, 12.28%) from Salt Lake soils, and then combined these with TerL proteins from viral references to construct a phylogenetic tree (Figure 2). As shown in Figure 2, the evolutionary branches of soil viruses are widely distributed across the phylogenetic tree, with the majority classified within the family *Siphoviridae* (*n* = 598), followed by *Myoviridae* (*n* = 94) and *Podoviridae* (*n* = 73). The novel soil viruses associated with Yuncheng Salt Lake exhibit significant diversity, as evidenced by the multiple independent small branches formed by the identified viral sequences. These clades represent previously uncharacterized diversity and may be considered novel branches of the *Caudovirales*, highlighting the genomic diversity of phages in our virome. The phylogenetic distances observed among viruses within a single clade reflect a comparable evolutionary diversity of *Caudovirales* across all soil samples. The close phylogenetic relationships among these clades and the viruses identified in soils provide insights into potential host associations, which may extend to other unclassified *Caudovirales*.

**Figure 3:**
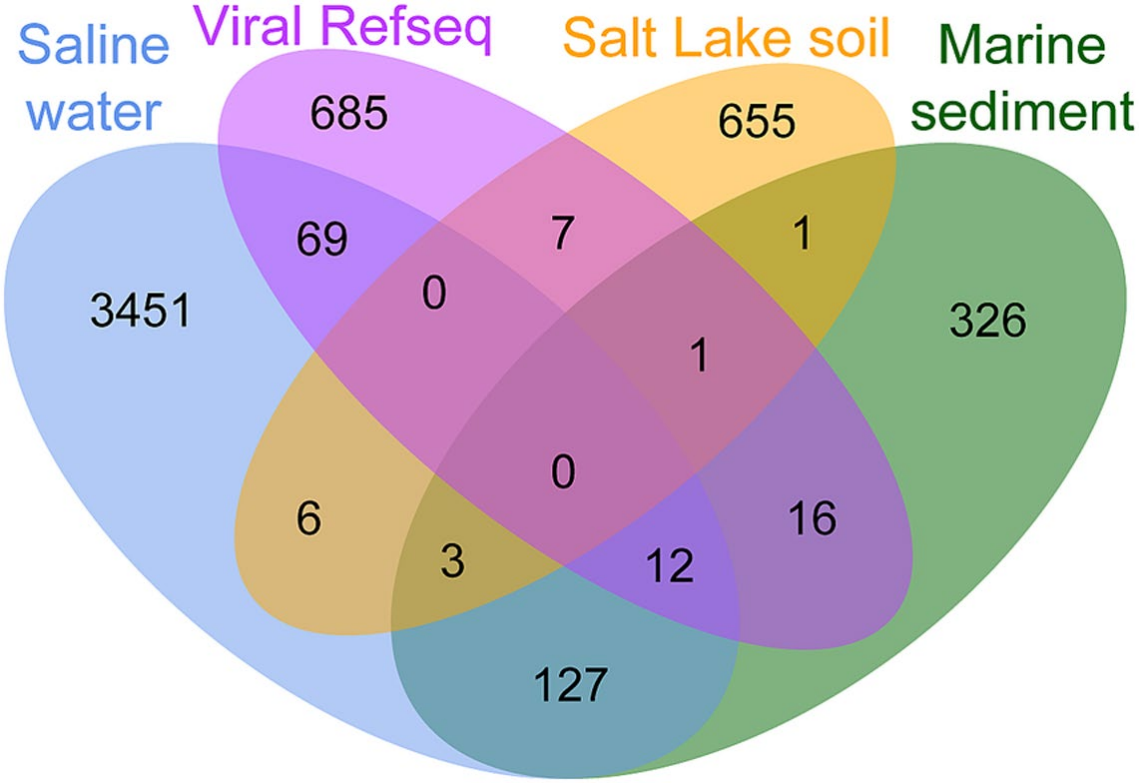
Venn diagram of shared viral clusters (approximately genus level) among the three environmental viral datasets and viral RefSeq.

In addition, vConTACT2 utilizes gene-sharing networks to cluster viral genomes into VCs at approximately the genus level. This approach allows for the exploration of relationships between vOTUs in Salt Lake soil and publicly available viral genomes from various ecosystems. The analysis identified a total of 5,358 VCs from vOTUs in Salt Lake soil, saline water, marine sediment, and Viral RefSeq, with only three VCs common across all ecosystems (Figure 3; Supplementary Table S2). The limited clustering among viral genomes suggests significant habitat specificity. In Salt Lake soil, 1,475 out of 3,362 vOTUs were clustered into 672 VCs, with 56.13% of which were not found in other ecosystems, indicating a considerable proportion of endemic viruses. Additionally, only nine VCs (43 vOTUs) were shared with saline water, five VCs (5 vOTUs) with marine sediment, and eight VCs (9 vOTUs) with viral RefSeq (Figure 3; Supplementary Table S3). These results suggest that Yuncheng Salt Lake likely harbors numerous unique viruses not identified in other environments, due to its distinct geographical location and saline conditions.

### 3.3 Distribution and abundance of viral communities

**Figure 4:**
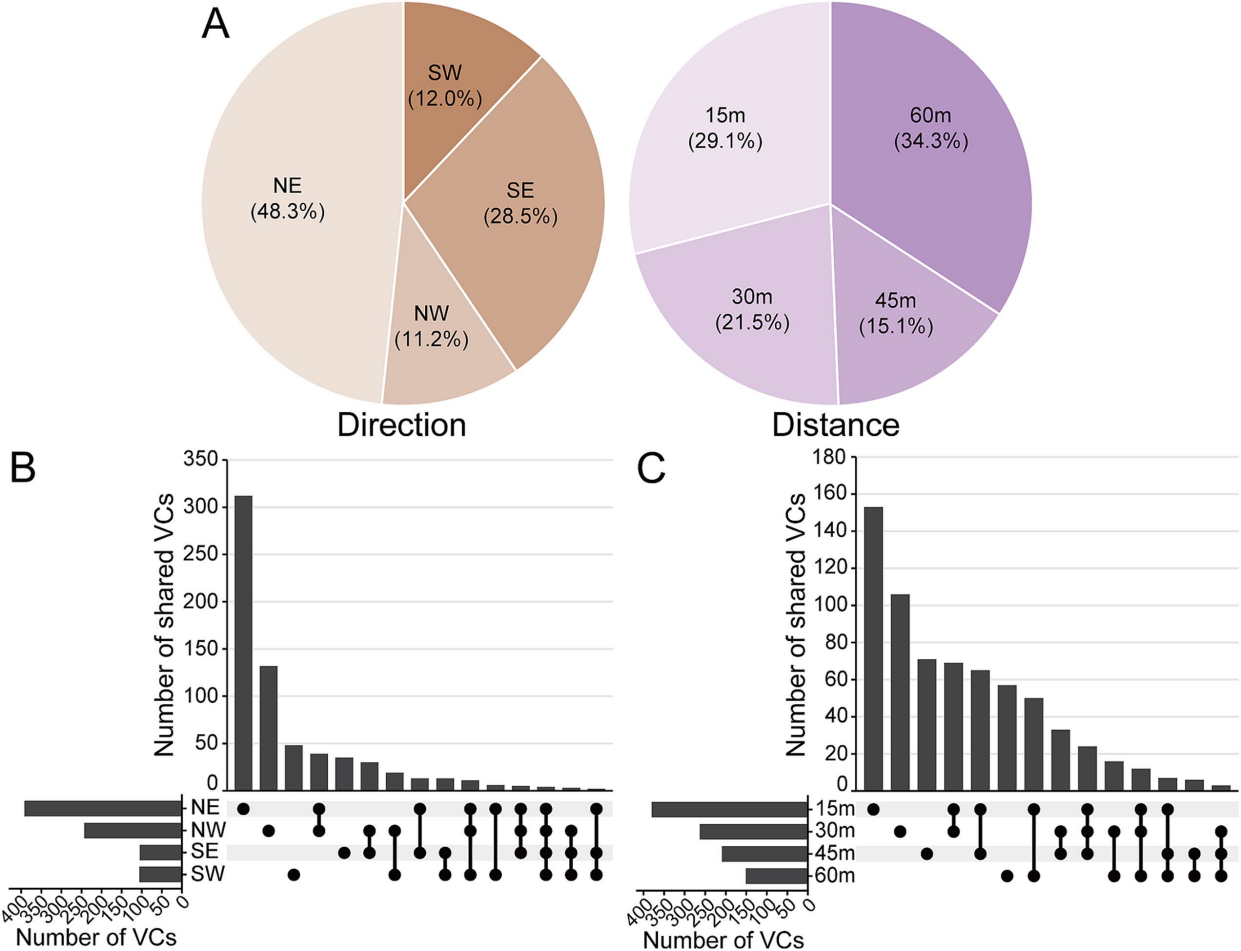
Distribution of vOTUs and viral clusters around Yuncheng Salt Lake soils. **(A)** Pie chart shows the percentage of the identified vOTUs in different diatance and direction around Salt Lake. UpSet plots show the shared number of the vOTUs in viral clusters among different direction **(B)** and distance **(C)**. NE, northeast; NW, northwest; SE, southeast; SW, southwest.

The abundance of vOTUs varies significantly by direction and distance from Salt Lake. Samples collected from 60 m accounted for 34.2% of the total vOTUs, while those from 15 m made up 29.12%. Notably, vOTUs in the NE direction represented nearly half of total at 48.3% (Figure 4A). The distribution ratios of vOTUs across families, based on direction and distance, were shown in Supplementary Figures S2, S3. Despite proximity to Salt Lake, there were limited overlap in viral clusters, indicating distinct geographical patterns in the surrounding Salt Lake soil. The number of viral clusters exclusive to the NE direction at 15 m was significantly higher than in other categories (Figures 4B,C).

**Figure 5:**
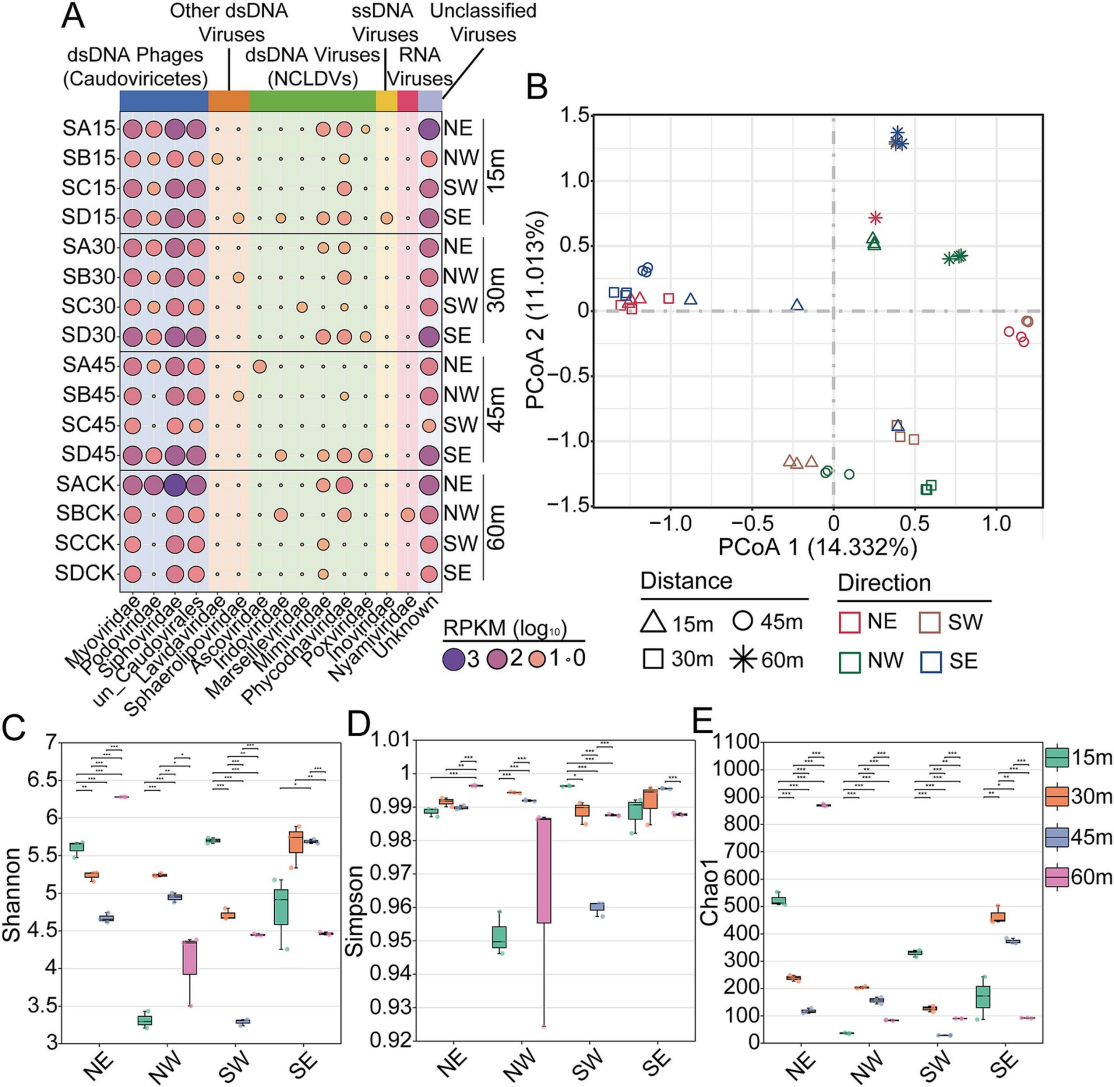
Structure and diversity of viral community in Salt Lake soils. **(A)** Relative abundance of viruses in each soil sample at the family level. **(B)** PCoA (principal coordinate analysis) based on Bray-Curtis of the viral communities in Salt Lake soils. Shannon **(C)**, Simpson **(D)**, and Chao1 **(E)** diversity of the viral communities. NE, northeast; NW, northwest; SE, southeast; SW, southwest.

To compare viral relative abundance profiles across samples, we calculated RPKM values for viral sequences classified at the family level (Figure 5A; Supplementary Table S4). Significant variations in the viral community composition of soils were observed based on directional and distance factors. Consistent with other studies on soil viromes, a substantial portion of the viral communities in Salt Lake soil comprised unclassified viruses, accounting for up to 56.81% of total viral reads (Figure 5A). In the order *Caudovirales*, *Siphoviridae* was the most dominant family, accounting for approximately 39% of the total, followed by *Myoviridae* at around 18% and *Podoviridae* at approximately 1.5%. NCLDVs made up 1.23% of the total viral abundance in Salt Lake soils, with *Phycodnaviridae* (0.72%) being the most abundant family, followed by *Mimiviridae* (0.24%). The abundance distribution of viruses within the *Phycodnaviridae* and *Mimiviridae* families showed a similar trend at the 15 m and 30 m sampling sites, but this trend reversed at the 45 m and 60 m sites. NCLDVs from these families have frequently been found in various components of ecosystems, including soil, water, ocean, and even human (Cheng et al., 2022; Schulz et al., 2022; Liang et al., 2024), but their ecological roles remain largely unexplored. Additionally, other dsDNA viruses, single-stranded DNA (ssDNA) viruses, and RNA viruses were also identified, with variations in their relative abundances across different sampling sites.

Additionally, principal coordinates analysis (PCoA) was employed to assess the degree of separation among viral communities, aiming to investigate the potential influence of geographic patterns on the distribution of viruses in Salt Lake soils. Distinct separation in viral taxonomic structure was observed in Salt Lake soil samples, with consistent distributions within samples collected from the same site (Figure 5B). Multivariate analysis methods separated samples according to various directions (Supplementary Tables S9, S10) and distances (Supplementary Tables S11, S12) (Adonis, *p* < 0.001). Dominant viral populations varied by distance; samples from 60 m were in the second quadrant, while others were spread across different quadrants. No clear directional distribution was observed, likely due to geographical isolation and nutrient availability variations in soils at different distances from the salt lake. The differences in viral communities observed at distinct sites in this study may be attributed to variations in *in situ* nutrient availability and/or differences in DNA recovery. Alternatively, these differences may also arise from variations in methods for metagenomic assembly and viral sequence extraction. In each of the four cardinal directions, the diversity scores at varying distances were corroborated by all three indices (Shannon, Simpson, and Chao1), as well as by the identified vOTUs, which were distinctly categorized based on distance (Figures 5C–E). In the northeast direction, the viral community diversity at 60 m was significantly greater than that observed in other soils, whereas in the northwest and southwest directions, the highest viral community diversity was recorded at the 30 m and 15 m sampling sites, respectively. The lowest viral diversity was contingent upon the sampling direction, with the minimum diversity recorded at 15 m in the northwest direction, 45 m in the southwest direction, and 60 m in the southeast direction. Furthermore, we aggregated all samples from various directions and distances to calculate the overall diversity index (Supplementary Figures S4, S5). The results indicate no significant differences in viral diversity across different distances, although the diversity in the northeast direction is slightly higher than in the other directions. Overall, our findings suggest that viral diversity is primarily characterized by sampling locations and is influenced by both distance and direction, with the extent of distance’s impact on viral diversity varying across different directions.

### 3.4 Virus-host linkages in salt lake soil

**Figure 6:**
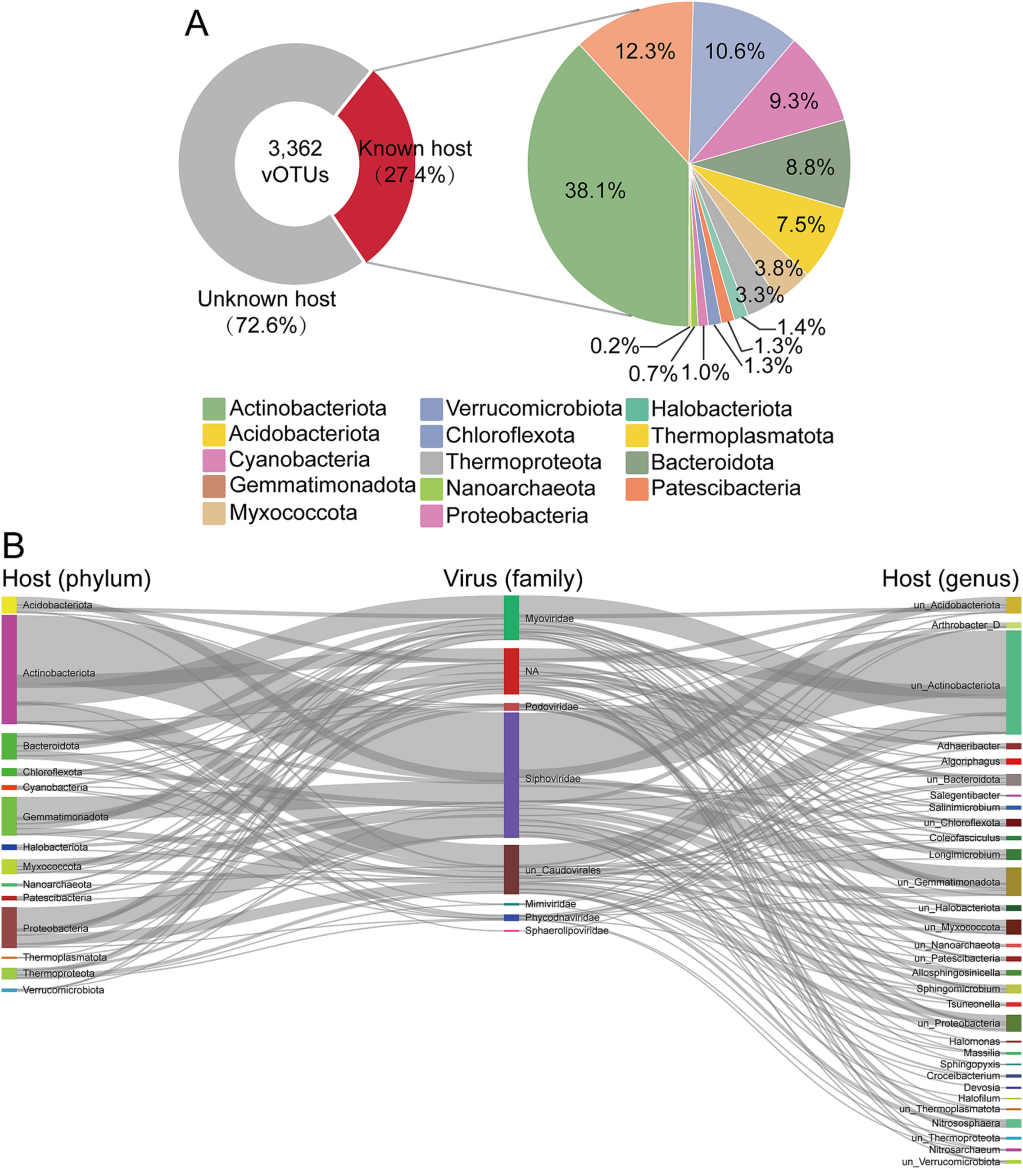
Virus-host linkages in soils. **(A)** The proportions of known host and unknown host (left) and the proportions of viral host at phylum level (right). **(B)** The linkages of different viral families with the taxonomy of predicted hosts at phylum level and genus level.

Viruses can significantly shape the diversity and structure of microbial communities by transforming lifestyle, lysing host cells, or coexisting with them. Which is crucial for comprehending the ecology and function of microbial communities (Suttle, 2007). To explore potential virus-host relationships, we correlated 237 MAGs with 3,362 vOTUs using four *in silico* methods (Paez-Espino et al., 2016). This effort successfully linked 921 vOTUs (27.4%) with 217 MAGs, forming virus-host pairs (Figure 5A; Supplementary Table S6). This proportion aligns with findings from other environments using similar methods (Emerson et al., 2018; Li et al., 2021). The prokaryotic hosts linked to vOTUs were classified into 14 phyla, with the majority of MAGs belonging to *Actinobacteriota* (29.03%, 63 of 217 MAGs), followed by *Proteobacteria* (14.75%, 32 of 217 MAGs) and *Gemmatimonadota* (13.36%, 29 of 217 MAGs). *Actinobacteriota* (376 out of 921 vOTUs) and *Gemmatimonadota* (121 out of 921 vOTUs) are the most frequently predicted host phyla (Figure 6A). The vOTUs with assigned host information predominantly belong to the class *Caudoviricetes* (Figure 6B). Linking virus-host taxonomy revealed that viruses within the same order or family often exhibit a broad host range. Notably, approximately 92.94% of the vOTUs were predicted to target specific hosts, with only 61 vOTUs associated with hosts from different prokaryotic phyla. This included 4 vOTUs spanning 3 phyla and 57 vOTUs spanning 2 phyla. The specificity of various viruses can result in significant differences in their host ranges. Viruses with a narrow host range are restricted to specific species or strains (Holmfeldt et al., 2007), whereas those with a broad host range can infect hosts across different kingdoms and may even simultaneously infect bacteria, archaea, or fungi, such as the generalist phages (Roux et al., 2019a). Additionally, multiple different viruses can co-infect the same host cell, as previously observed in marine bacteria (Roux et al., 2014). This observation supports existing theories and prior research indicating that most viruses exhibit a restricted host range.

### 3.5 Functional profiles and auxiliary metabolic genes of viruses

**Figure 7:**
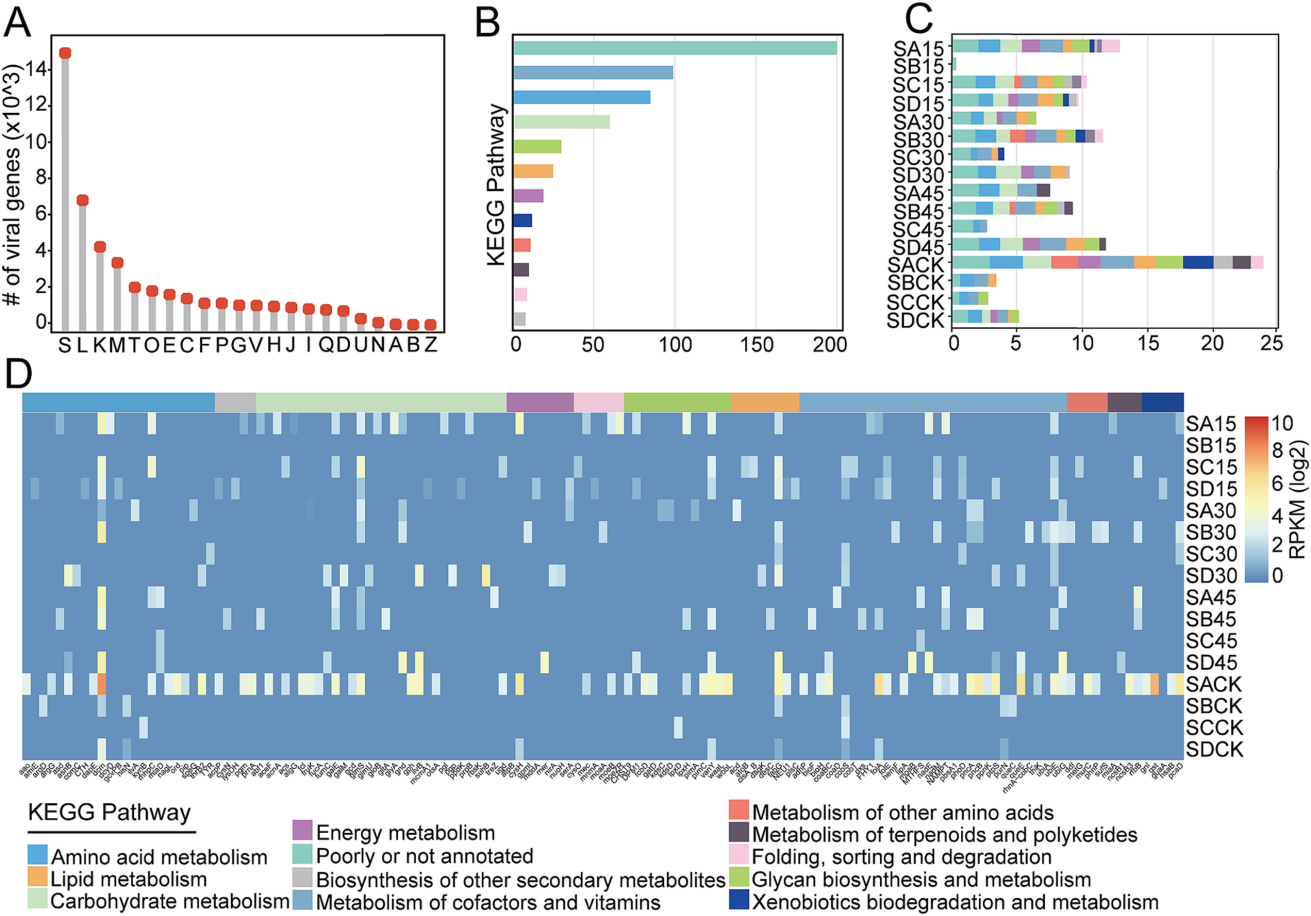
Functional genes and AMGs encoded by soil viruses. **(A)** The number of viral functional genes categorized by COG classes. The detailed descriptions of COG function classes were: ‘S’ (Function unknown), ‘L’ (Replication, recombination and repair), ‘K’ (Transcription), ‘M’ (Cell wall/membrane/ envelope biogenesis), ‘T’ (Signal transduction), ‘O’ (Posttranslational modification, protein turnover, chaperones), ‘E’ (Amino acid transport and metabolism), ‘C’ (Energy production and conversion), ‘F’ (Nucleotide transport and metabolism), ‘P’ (Inorganic ion transport and metabolism), ‘G’ (Carbohydrate transport and metabolism), ‘V’ (Defense mechanisms), ‘H’ (Coenzyme transport and metabolism), ‘J’ (Translation), ‘I’ (Lipid transport and metabolism), ‘Q’ (Secondary metabolites biosynthesis, transport and catabolism), ‘D’ (Cell cycle control, cell division, chromosome partitioning), ‘U’ (Intracellular trafficking, secretion, and vesicular transport), ‘N’ (Cell motility), ‘A’ (RNA processing and modification), ‘B’ (Chromatin structure and dynamics), ‘Z’ (Cytoskeleton). **(B)** The number of viral AMGs involved in different metabolic categories. **(C)** Relative abundance of the viral AMGs in different metabolic categories. **(D)** Relative abundance of the viral AMGs across Salt Lake soil samples. KEGG metabolic categories are colored according to the legend.

To investigate the potential functions of the virus in Salt Lake soil, we utilized the eggNOG database to assign functions to all 121,019 predicted genes in the viruses (Supplementary Table S7). As a result, 64,134 genes from the total viral gene pool were annotated to known orthologous groups, with 23.3% (*n* = 14,928) classified as functionally unknown (Figure 7A). Among the identified functional genes, a significant proportion is associated with viral replication and transcription, exhibiting high relative abundance, particularly within the categories of “replication, recombination and repair (L),” “transcription (K)” and “cell wall/membrane/envelope biogenesis (M).” Notebly, 1.9% (*n* = 1,196) of the all annotated functional genes in viruses were linked to “carbohydrate transport and metabolism (G)” (Figure 7A), a finding also observed in mangrove sediment (Jin et al., 2019) and plateau salt lakes (Gu et al., 2021).

Viruses that carry host-derived AMGs can express these genes within the host cells after infection, thereby manipulating the metabolic processes of the host (Breitbart et al., 2007). Due to environmental selective pressures, those AMGs that help maintain and/or enhance the adaptability of the viruses or their hosts are retained in the genomic sequence (Breitbart et al., 2018). To elucidate the potential roles of viruses in influencing critical biogeochemical cycles, a total of 568 viral AMGs within 316 vOTUs, representing 9.4% of all vOTUs, were identified across 11 pathways as defined by KEGG that are linked to targeted processes, including carbon, nitrogen, sulfur, and phosphorus cycles. This identification was achieved through the application of DRAM-v and VIBRANT (Supplementary Table S8). Among these, the number of AMGs carried by viruses varied from 1 to 14, with the vast majority of viral sequences containing fewer than 4 AMGs (Supplementary Table S8). The viruses predominantly encoded AMGs related to cofactor and vitamin metabolism (a.g. pantothenate and CoA biosynthesis, and folate biosynthesis intermediates), followed by amino acid metabolism (e.g., methyltransferases, aminotransferases, asparagine synthase), and carbohydrate metabolism (primarily involving the metabolism of various nucleotide, sugars and oligosaccharides) (Figure 7B; Supplementary Table S8).

Through the analysis of the composition and abundance of viral AMGs in Salt Lake soil samples, the results indicated that viruses in these soils harbored a significant number of AMGs with diverse functional roles (Figures 7C,D). Notably, AMGs associated with cysteine and methionine biosynthesis, particularly DNA [cytosine-5]-methyltransferase 1 (*dmc*) were found to be overrepresented in the viral genomes of the soil samples when compared to other metabolic pathways. These metabolic functions have been documented as being enriched in marine viruses (Enav et al., 2014), suggesting that the selective pressures present in various habitats can result in a consistent acquisition of AMGs. Under specific environmental conditions, AMGs that entered host cells through viruses might compensate for the host’s metabolic deficiencies or introduce new metabolic pathways. This alteration of the host’s metabolic processes enabled the host to adapt to the current environment, as observed in marine ecosystems (He et al., 2017). After the virus carrying AMGs entered the host cell, certain AMGs could redirect the host’s metabolism to enhance resource supply or recycle intracellular nucleotides for viral replication (Lavigne et al., 2013). Additionally, some AMGs may provide the host with defense mechanisms for protection, while others, such as DNA methyltransferases, can help the virus evade host restriction endonucleases (Murphy et al., 2013).

Yuncheng Salt Lake is characterized by the presence of sodium sulfate (Na_2_SO_4_) (Zhou et al., 2019). Within this ecosystem, both sulfur oxidation and sulfate reduction played crucial roles in the sulfur cycling process (Liu et al., 2022). Among the 568 viral AMGs identified, 21 were annotated as being involved in sulfur metabolism. Notably, eight of these AMGs encode phosphoadenosine phosphosulfate reductases (c*ysH*), which are integral to assimilatory sulfate reduction. Recent studies have also reported the presence of AMGs coding for *cysH* in viruses obtained from deep-sea sediments (Zhou et al., 2022; Yu et al., 2024), salt lakes (Gu et al., 2021), and seawater (Li et al., 2021; Zhou et al., 2022). The remaining 13 viral AMGs were associated with the sulfur relay system, which included sulfur transferases such as sulfate adenylyltransferase (*cysN*) and sulfur-carrier protein adenylyltransferase (*moeZR*), as well as sulfur-carrier proteins like *moaD* and *mec* (CysO sulfur-carrier protein-S-L-cysteine hydrolase). Furthermore, it has been proposed that the sulfur relay system contributed to microbial tolerance against acid stress (Cao et al., 2019), heavy metals (Gang et al., 2019), and organic solvents (Liu et al., 2012). Consequently, these AMGs may enhance the adaptability of their hosts to various stress conditions, thereby providing a competitive advantage in salt soil environments characterized by significant fluctuations in temperature and chemistry. Additionally, viral AMGs that facilitated the metabolism of carbon (C), nitrogen (N), and phosphorus (P) were also identified in Salt Lake soil viruses. In terms of carbon metabolism, one viral AMG encoding pyruvate phosphate dikinase (*ppdk*) was discovered, which was involved in the reductive tricarboxylic acid (rTCA) and dicarboxylate/4-hydroxybutyrate (DC/4-HB) cycles (Hügler and Sievert, 2011), represented the primary pathways for dark carbon fixation by deep-sea microbes (Hügler and Sievert, 2011). Regarding nitrogen metabolism, virus-encoded *nirA* (ferredoxin-nitrite reductase), associated with dissimilatory nitrate reduction, along with numerous genes related to organic degradation and synthesis, were also detected. We also found several virus-encoded phosphorus metabolism AMGs, including *phoD*, *gph* and *pgp* genes related to organic phosphoester degradation. In particular, organophosphates are recognized as emerging contaminants that pose a significant risk to human health (Silva and Baltrusaitis, 2021). The presence of viruses carrying phosphorus metabolism AMGs underscored the essential role of viruses in phosphorus metabolism. Moreover, the most abundant AMG identified in the metagenomes of Salt Lake soil was DNA cytosine methyltransferase (DNMT1, *dcm*). However, virus-encoded *dcm* genes have been found in metagenomes from various environments, suggesting that they performed central functions in the viral life cycle (Waite et al., 2018). Consequently, the presence of viral AMGs that directly mediated the metabolic processes of host cells might also exert an indirect influence on biogeochemical cycles (Zimmerman et al., 2020). This suggests that viruses played a crucial role in regulating the metabolism of host cells and, in turn, affected biogeochemical cycles within this particular region.

## 4 Conclusion

Salt lakes harbored a diverse array of unique microorganisms and their associated viruses; however, the role of viruses in these ecosystems—particularly in their interactions with microbial hosts regarding aspects such as microbial mortality, ecological functions, and co-evolution—has largely remained underexplored. This study revealed the novelty, richness, and diversity of prokaryotic viruses harbored in soils collected from Yuncheng Salt Lake, as determined through metagenomic analysis. The viral communities exhibited distinct spatial separation in the soils surrounding Yuncheng Salt Lake. This phenomenon appeared to be influenced by variations in salt ion concentration and nutrient availability, which, in turn, shaped the structure of the prokaryotic host communities across different sampling sites. From the same metagenomic data, the identified viral sequences were *in silico* associated with the reconstructed prokaryotic MAGs, and a significant proportion of vOTUs could be linked to the host MAGs. It is worth noting that many of the putative soil viral microbial hosts belong to taxonomic groups that do not have cultured representatives. These findings enhanced our understanding of the diversity of halotolerant archaeal viruses, particularly those that infected important archaeal lineages present in halotolerant microorganism, such as *Halobacteriota*, *Nanoarchaeota*, and *Thermoproteota*. The potential presence of lytic viral communities in the salt lake soils might contribute to the high microbial density observed in this otherwise nutrient-deficient environment through the viral shunt mechanism, which influenced microbial mortality and biomass turnover. In addition, 316 soil viruses carried 568 AMGs that performed various functions related to viral replication, as well as participated in the elemental cycling of carbon, sulfur, phosphorus, and other elements. These genes may compensate for the host’s metabolic pathways during viral infections or introduce new metabolic pathways to enhance the metabolic processes of prokaryotic hosts, potentially impacting the biogeochemical processes within microbial communities. Salt lakes served as natural reservoirs of halotolerant prokaryotic diversity and were vital oases for the activity of viruses associated with these microorganisms. Considering that the data were obtained from a public database, information on the environmental factors associated with the samples is lacking. This study aimed to explore the diversity, geographical distribution, and potential ecological functions of viruses in soils surrounding Yuncheng Salt Lake. Future research on salt lake should involve re-sampling and measuring key environmental parameters such as temperature, pH, salinity, and nutrient levels. These measurements should be integrated with metatranscriptomic analysis and key gene expression studies to better understand the environmental factors influencing the viral community and the functions of virus-encoded AMGs in this environment.

This study did not investigate the co-evolution of viruses with microbial communities inhabiting salt lakes and their surrounding environments, particularly concerning their potential roles in horizontal gene transfer, as well as the seasonal variations in virus-host interactions. These aspects necessitate further research and exploration. Future research should focus on the high-throughput cultivation of salt lake viruses to investigate the interactions between these viruses and their hosts. The findings could offer valuable insights into the metabolic potential of microorganisms and viruses in salt lakes regarding nutrient cycling, the efficiency of viral dispersal, and the dynamics of virus-host interactions. Given that only a small portion of the identified vOTUs can be classified, and many predicted to be infected belong to poorly characterized taxa, there remains a significant gap in our understanding of the factors influencing the biogeochemistry of salt lake soils—including anthropogenic influences—as well as the factors affecting the metabolism and functional adaptations of viruses and microorganisms in these environments. These topics will be important areas for future investigation.

## Data Availability

The raw reads of metagenomes were deposited in GenBank under BioProject number PRJNA861910. The original contributions of this study are included in the article/Supplementary material. Other data will be made available on request.

## Footnotes

^1^ https://github.com/feargalr/Demovir

^2^ https://github.com/wwood/CoverM

^3^ https://www.ncbi.nlm.nih.gov/labs/virus/

^4^ https://www.chiplot.online/

## Supplementary material

SUPPLEMENTARY FIGURE S1 Histogram showing the distribution of viral genome size.

SUPPLEMENTARY FIGURE S2 The pie chart shows the percentage of different virus families in different directions. NE, northeast; NW, northwest; SE, southeast; SW, southwest.

SUPPLEMENTARY FIGURE S3 The pie chart shows the percentage of different virus families in different distances.

SUPPLEMENTARY FIGURE S4 The Shannon, Simpson, and Chao1 diversity indices of viruses in Salt Lake soils across various directions. NE, northeast; NW, northwest; SE, southeast; SW, southwest.

SUPPLEMENTARY FIGURE S5 The Shannon, Simpson, and Chao1 diversity indices of viruses in Salt Lake soils across various distances.
