# Supplementary figures and images for: Characterization, diversity, and biogeochemical potential of soil viruses inhabiting in Yuncheng Salt Lake
Source: Front Microbiol. 2025 May 7;16:1597514. doi: 10.3389/fmicb.2025.1597514 (PMC12092443; doi:10.3389/fmicb.2025.1597514)

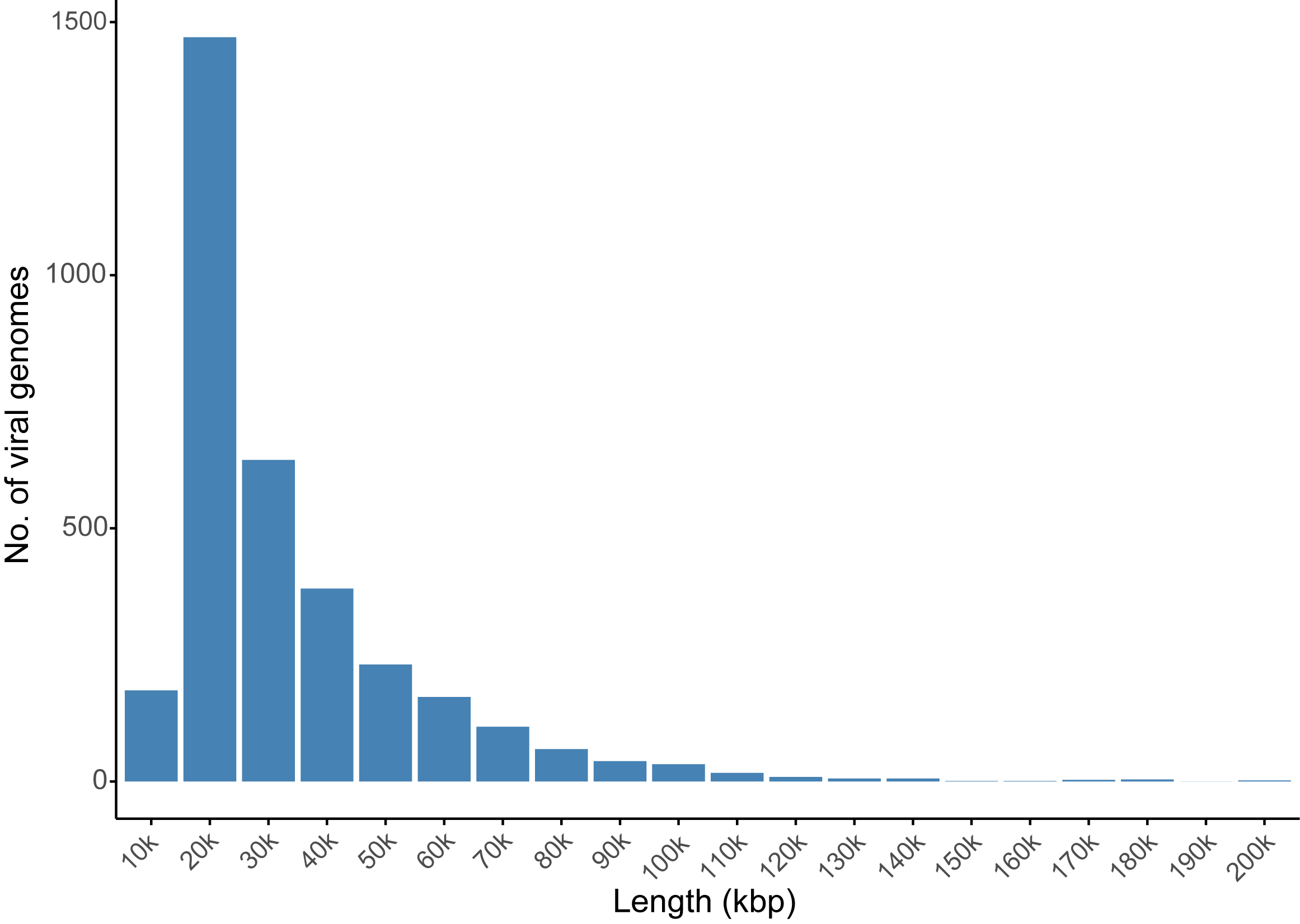

Supplement: SUPPLEMENTARY FIGURE S1 — Histogram showing the distribution of viral genome size. [file Image_1.TIF]

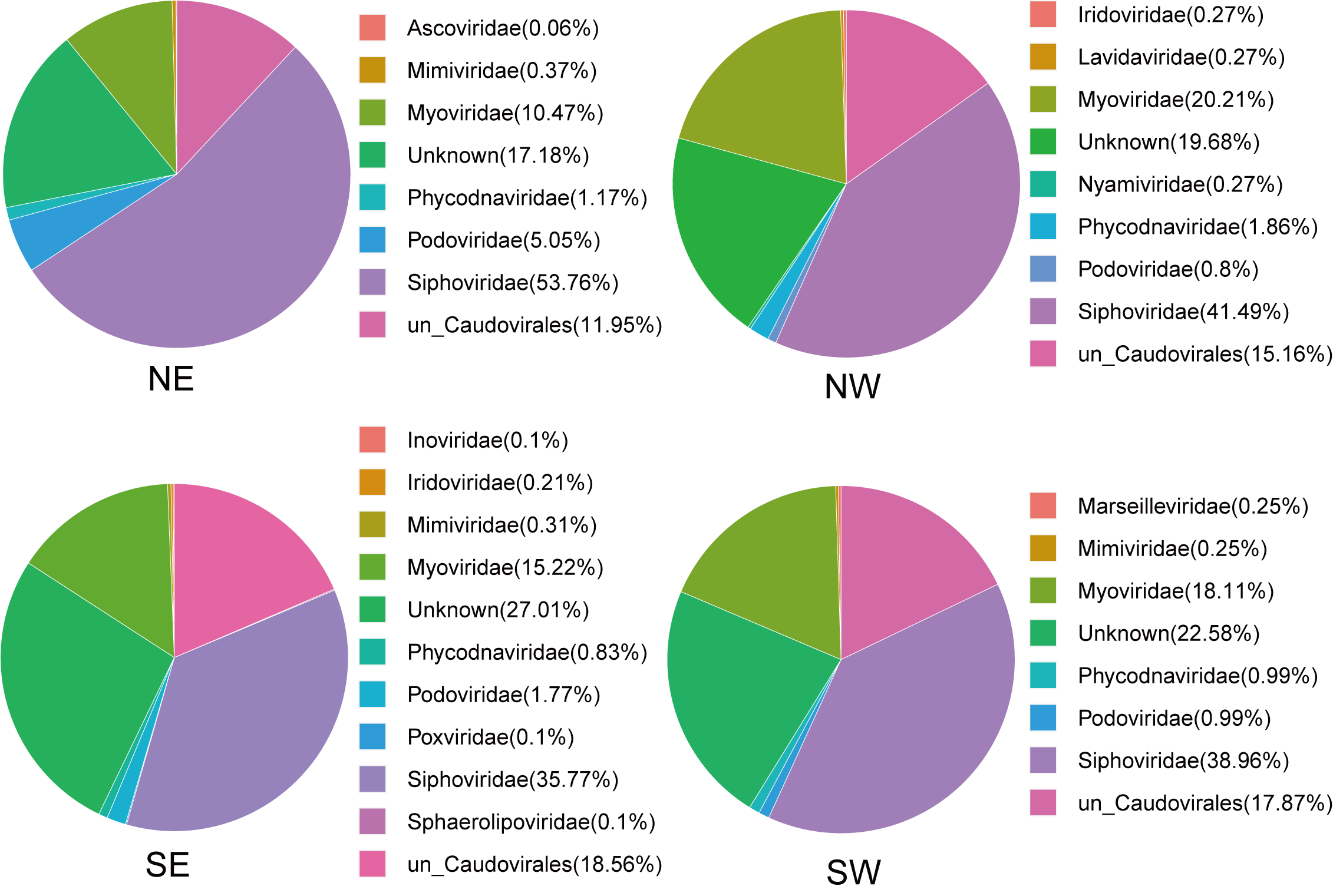

Supplement: SUPPLEMENTARY FIGURE S2 — The pie chart shows the percentage of different virus families in different directions. NE, northeast; NW, northwest; SE, southeast; SW, southwest. [file Image_2.TIF]

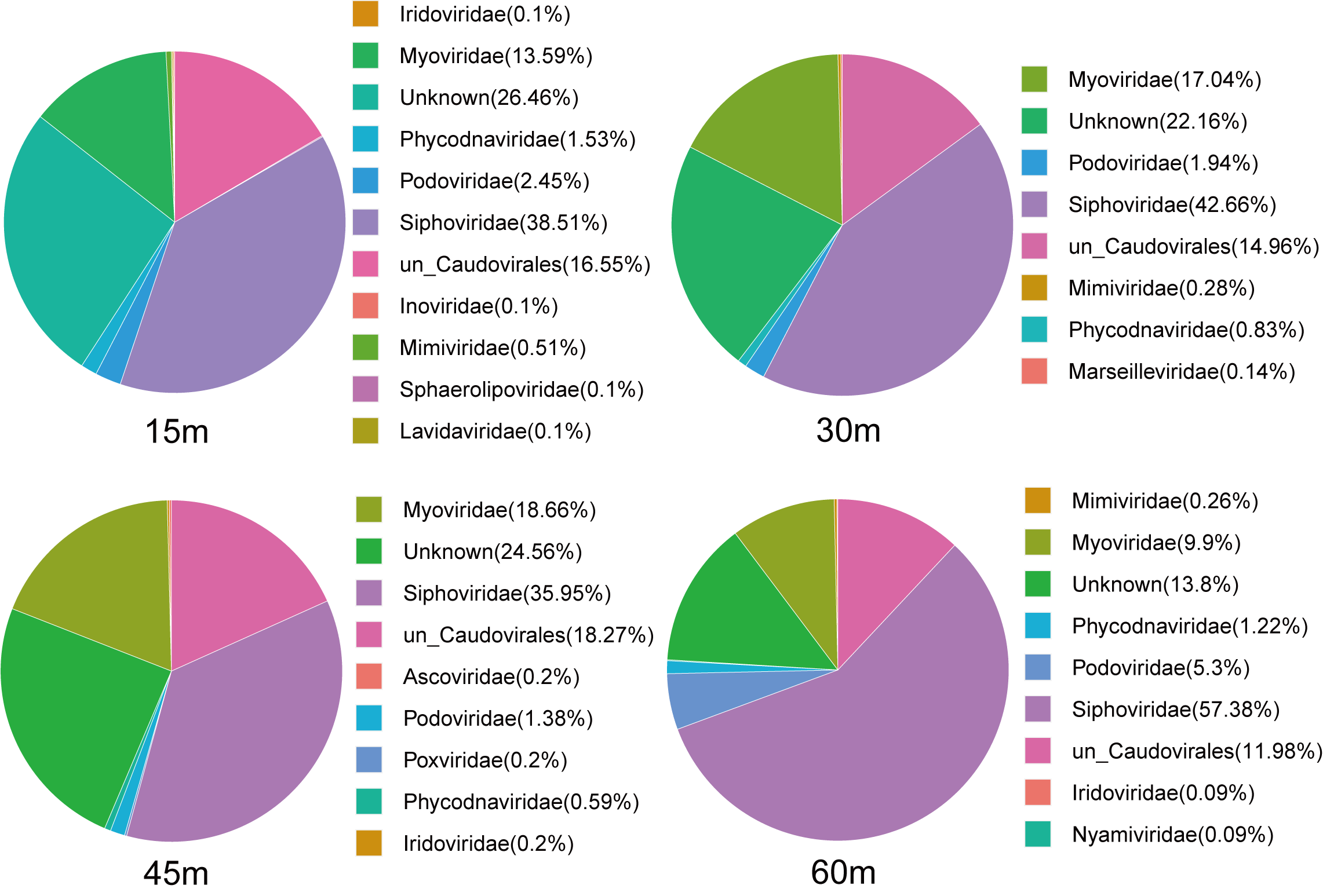

Supplement: SUPPLEMENTARY FIGURE S3 — The pie chart shows the percentage of different virus families in different distances. [file Image_3.TIF]

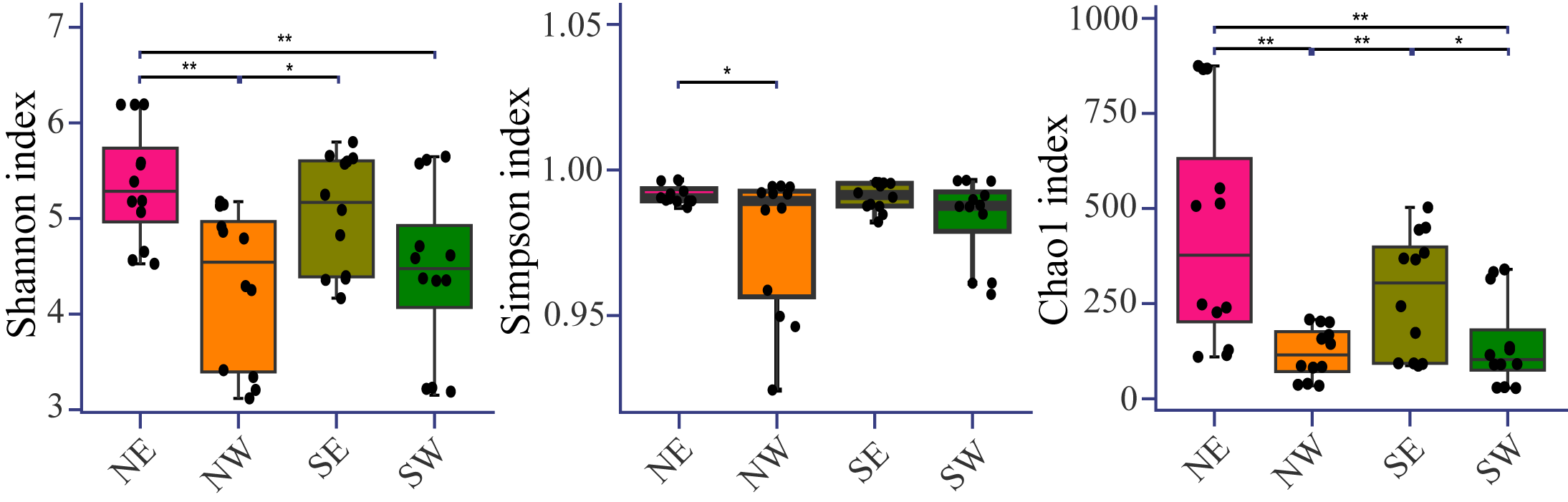

Supplement: SUPPLEMENTARY FIGURE S4 — The Shannon, Simpson, and Chao1 diversity indices of viruses in Salt Lake soils across various directions. NE, northeast; NW, northwest; SE, southeast; SW, southwest. [file Image_4.TIF]

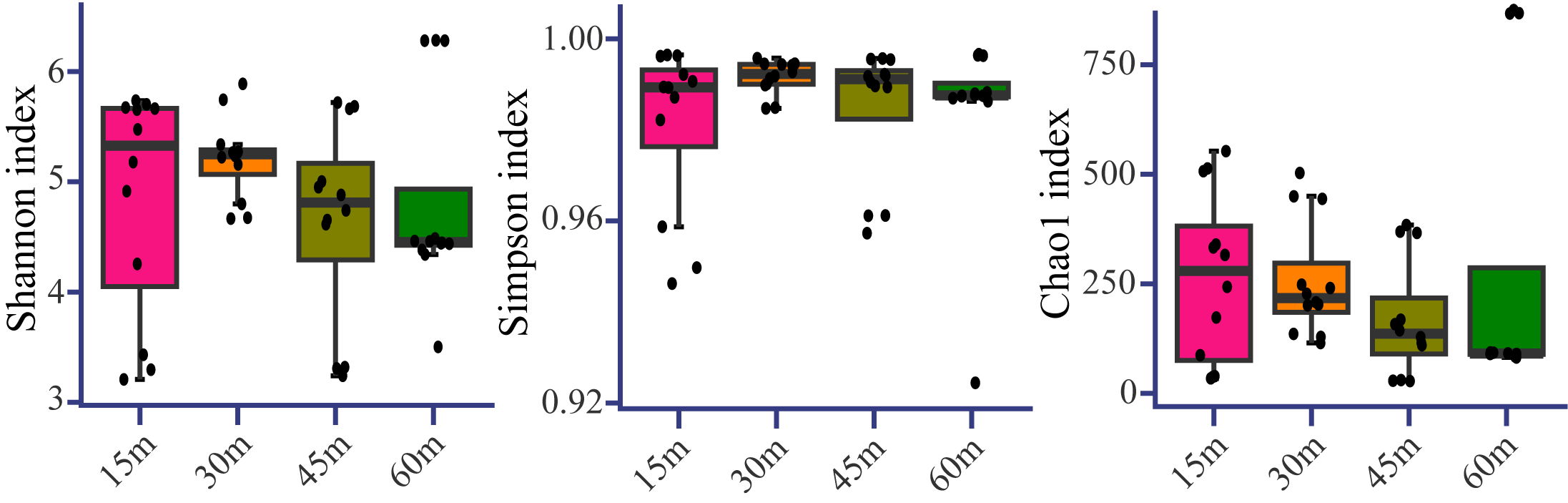

Supplement: SUPPLEMENTARY FIGURE S5 — The Shannon, Simpson, and Chao1 diversity indices of viruses in Salt Lake soils across various distances. [file Image_5.TIF]
